# Laparoscopic treatment of a large simple hepatic cyst misinterpreted as hydatid

**DOI:** 10.1093/jscr/rjae780

**Published:** 2024-12-11

**Authors:** Svetlana Shumarova

**Affiliations:** Department of Surgery, University Hospital “Aleksandrovska” Sofia, Bulgaria, Medical University, 1 Georgi Sofijski Blvd, 1431 Sofia, Bulgaria

**Keywords:** liver cyst, simple cyst, hydatid cyst

## Abstract

In addition to becoming the gold standard for surgical treatment of a number of diseases, laparoscopy is sometimes needed as a diagnostic method to differentiate one disease from another. We present a case of a 75-year-old man with an incidentally found liver cyst, which was suspected to be hydatid. We performed a laparoscopic deroofing and a simple cyst was found, which diagnosis was confirmed the final pathological analysis of the cyst wall.

## Introduction

Simple liver cysts are serous fluid-filled cavities that are thin-walled and arise from aberrant bile duct cells. Common cystic lesions that present diagnostic challenges are hydatid cysts, simple cyst/ polycystic liver, and intrahepatic cystadenoma [1]. Correct preoperative diagnosis is essential, as the therapeutic approach is different [1]. The simple liver cyst may be asymptomatic and may found on incidental ultrasound examinations on another occasion. Pain is present in a significant percentage of cases with simple liver cysts depending on location and size [2–6]. We present a clinical case of an asymptomatic liver cyst suspicious for hydatid.

## Case report

**Figure 1:**
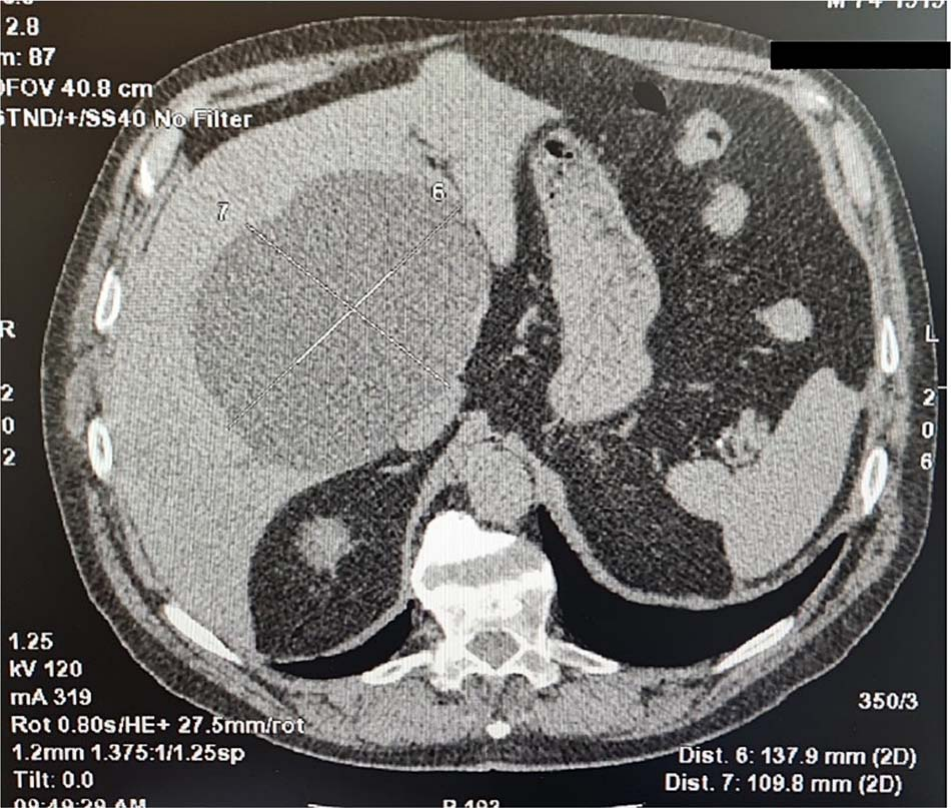
Computed tomography imaging of the liver cyst.

A 75-year-old man was admitted to a surgery clinic after detected a large cystic formation in the right lobe of the liver with prophylactic ultrasound (US) examination. For two years, the cyst was tracked using computed tomography (CT) (Fig. 1) and gradually increased in size from 10 cm to 13 cm. An ELISA method was performed to differentiate from a hydatid cyst, which was positive. Computed tomography described a large cystic formation in the right lobe of the liver involving segments IV, V, VIII measuring 137.9 mm to 109.8 mm with smooth contours. Laboratory findings were normal. A diagnosis of hydatid cyst was made due to the positive ELISA method and the patient was offered laparoscopic surgery.

**Figure 2:**
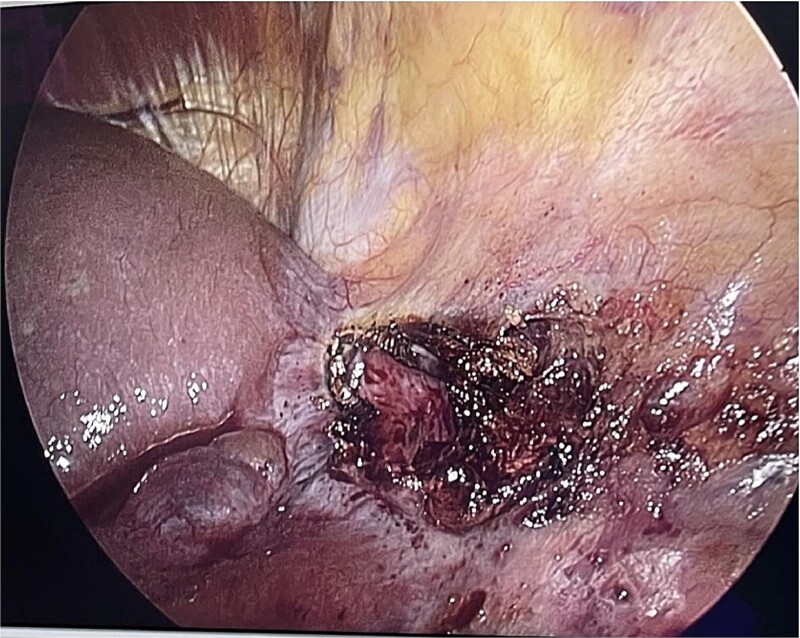
Anterior surface of the liver cyst-(laparoscopic view).

**Figure 3:**
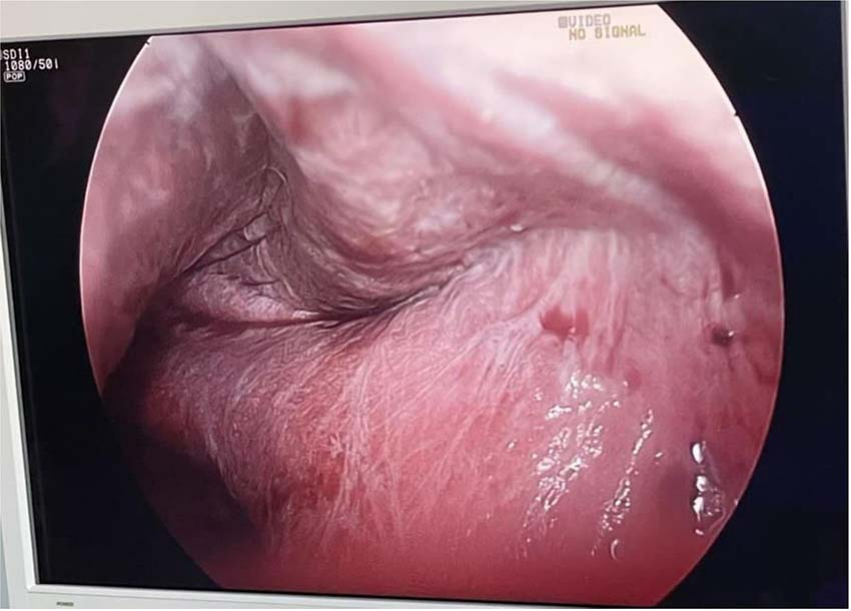
Laparoscopic view of the cyst inside.

The patient was in the supine position (American position) and a Veress needle was inserted through a subumbilical skin incision and a pneumoperitoneum was created to 13 mmHg, after which a 10 mm optic trocar was inserted. Under optical control, another 5 mm trocar was inserted subxiphoidally and one 5 mm on the right mid-axillary line subcostally. Intraoperatively, a small whitish ribbon-like area was visualized in the area of the IVB segment with a length of ⁓3 cm/d. Using LigaSure, the Falciform ligament was resected for better visualization. Using a hook, the capsule of the cyst was opened and serous fluid was evacuated, which proved to be a simple cyst (Fig. 2). Cyst walls were excised down to the parenchyma using LigaSure. The cyst was visualized inside to rule out communication with the bile duct (Fig. 3). Due to the depth and intraparenchymal location, a drain was placed inside, which was removed on the 20th postoperative day until the drainage from the drain stopped completely. The total operative time was 40 min and the patient was discharged with a drain on the 2nd postoperative day. Pathomorphological examination of the removed cyst wall proved a simple cyst.

## Discussion

A recent analysis by Kersik *et al*. [4] found a higher incidence of simple liver cyst in men, with a male: female ratio of 1:3.2. The average age of the patients is 72 years with a higher percentage of localization on the right, compared to the left site [4]. The results are similar in the cases presented by us in Table 1. Karan *et al*. [1] included in an analysis 65 cases of liver cysts, of which 67.7% (n = 44) had hydatid cysts, 20% (n = 13) simple cyst, and 12.3% (n = 8) with biliary cystadenoma. Accurate dedifferentiation is necessary for proper planning for a precise therapeutic approach. There is insufficient data regarding the role of some tumor markers in differentiation a simple cyst from cystadenoma or cystadenocarcinoma. The marker CA 19–9 is a tumor-associated antigen and is increased in cases of cancer of the colon, stomach, biliary tract and pancreas, but some authors report an increase in serum levels also in simple cysts [2, 3, 5, 6], which excludes at this stage its possibility to play a role in the differential diagnosis. The same is true for CEA marker, which is also found to be elevated in simple liver cysts [3, 5]. A recent report by Zhang [3] found elevated values of biomarkers protein induced by vitamin K absence (PIVKA), and the cause may be due to the compression of a large liver cyst, which damages hepatocytes.

**Table 1:** Clinical cases.

Reference	Journal	Year	Age, Years	Gender	Clinical presentation	Size, cm	Anatomical site	Imaging	Test for hydatidosis	CEA	AFP	PIVKA-II	CA19–9 N < 27 U/mL	CA125	ASAT	Bilirubin	ALAT	CA72.4	Access	Operation	Drain
Letizia Zurli [2]	Clinical Journal of Gastroenterology	2021	67	female	pain	25.00	right	US,CT	negative	normal	normal	–	increased	–	–	–	–	normal	Laparoscopic	fenestration	Yes
Jia-Wei Zhang [3]	World J Gastrointest Surg	2020	84	female	pain	20.00	right	CT	–	–	–	increased	increased	increased	increased	increased	increased	–	–	Percutaneous drainage+ polycinnamol sclerosing	Yes
Taro Ikeda [7]	Journal of Surgical Case Reports	2023	76	female	asymptomatic	24.00	right	CT, MRI	–	normal	–	–	normal	–	–	–	–	–	Laparoscopic	deroofing	Yes
Alessia Kersik [4]	Eur Surg	2023	63	female	pain	21.00	right	US, MRI	negative	normal	normal	–	normal	normal	–	–	–	–	Laparoscopic	deroofing	–
Xu-Xia He [8]	World J Gastrointest Surg	2022	39	female	icterus	13.70	hilum	US,MRI	–	increased	–	–	–	–	increased	increased	increased	–	–	percutaneous catheter and sclerotherapy	Yes
Ryosuke Mizukami [5]	Journal of Surgical Case Reports	2024	72	man	pain	17.00	right	CT,MRI	–	increased	normal	–	increased	increased	–	–	–	–	Laparoscopic	fenestration	–
Kengo Yoshitomi [6]	Case Reports in Gastrointestinal Medicine	2024	74	female	pain	8.00	left	US,CT	–	normal	–	–	increased	–	–	–	–	–	–	transgastric drainage	–

Ultrasound is a useful and non-invasive method for the diagnosis of a simple liver cysts, which may be the only one for differentiation form abscesses, malignant tumors, hemangiomas and hamartomas [9]. Contrast-enhanced CT is negative for the internal structures of cystic lesions according to Shimizu *et al.* [9], and magnetic resonance imaging (MRI) defines a spherical lesion with high signal intensity on T2-weighted images and low signal intensity on T1-weighted images without contrast enhancement.

Most of the authors are of the opinion that asymptomatic liver cysts do not require treatment, but only observation, but it should be taken into account that there is an occurrence of communication of the cyst with the biliary tree [7], which would be a reason for rapid growth with risk for rupture [5]. With the advent of minimally invasive treatment, laparoscopy has become the preferred method of treatment with proven effectiveness [2, 4, 5, 7]. A literature review by Kersik *et al.* [4] found only 22 publications detailing the surgical procedure at 20.8% laparoscopic deroofing was combined with omentopexy (n = 8), argon plasma coagulation (n = 4), or was preceded by ethanol sclerotherapy (n = 4). There is still no clear consensus in the literature regarding the actual efficacy of these techniques in terms of recurrence rates [4]. Percutaneous ultrasonographic guided aspiration is an effective method for immediate symptom relief in large liver cysts [7, 8], but according to Shimizu *et al.* [9] recurrence and new filling of the cyst is inevitable.

## Conclusion

Laparoscopy, in addition to being a good method with proven therapeutic effectiveness, can also be a good diagnostic method in case of doubt in differentiating a simple cyst from a cystadenoma, cystadenocarcioma, or hydatid cyst.
